# Multimorbidity patterns and early signals of diabetes in online communities

**DOI:** 10.1093/jamiaopen/ooaf049

**Published:** 2025-05-30

**Authors:** Ching Jin, Zhen Zhu

**Affiliations:** Centre for Interdisciplinary Methodologies, University of Warwick, Coventry CV4 7AL, United Kingdom; Kent Business School, University of Kent, Canterbury CT2 7FS, United Kingdom

**Keywords:** multimorbidity, early signals, diabetes, online communities, network science, data science

## Abstract

**Objectives:**

This study aims to explore multimorbidity patterns associated with diabetes by analyzing user engagement in online diabetes support communities and their interactions with other disease-related communities. Additionally, it seeks to assess whether early signals of diabetes can be detected through online engagement data.

**Materials and Methods:**

We collected Reddit data for 3 primary diabetes-related subreddits (“diabetes,” “diabetes_t1,” and “diabetes_t2”) and 88 other disease-related subreddits from 2008 to 2024. A bipartite network was constructed linking users to subreddits, which was then transformed into a weighted multimorbidity network. Significant links were identified using a statistical threshold to ensure meaningful connections between subreddits. Additionally, we analyzed user engagement timelines to identify potential early signals of diabetes.

**Results:**

Diabetes is strongly linked to mental health conditions (such as depression, anxiety, and ADHD) and weight management discussions. Other notable associations include autoimmune diseases, chronic pain, gastrointestinal disorders, and reproductive health issues. Early signals of type 2 diabetes were detected in mental health, obesity, and pregnancy conditions, but no significant early indicators were found for type 1 diabetes.

**Discussion:**

This study is the first large-scale empirical analysis of multimorbidity patterns and early signals of diabetes in online communities. The findings reinforce the known multimorbidity of diabetes, particularly its ties to mental health and obesity. The presence of early signals suggests that social media data could help identify individuals at risk before diagnosis, offering opportunities for early intervention.

**Conclusion:**

Our findings demonstrate that social media data can reveal both multimorbidity patterns and early signals of diabetes, offering insights beyond traditional health records. As digital health data continue to grow, effectively leveraging these resources will become increasingly important for advancing diabetes prevention and management.

## Background and significance

Diabetes is a complex chronic disease that frequently coexists with other health conditions, known as multimorbidity,^1^^,^^2^ including obesity,^3^ cardiovascular disease,^4^ kidney disease,^5^ and mental health disorders such as depression and anxiety.^6–8^

Diabetes diagnosis and care are typically managed through traditional health systems. However, these systems can only capture patient information through limited interactions and timeframes.^9^ Moreover, they struggle to address the complex and evolving nature of multimorbidity associated with chronic conditions.^10^ Mental healthcare poses even greater challenges in terms of generating and maintaining medical records than physical healthcare due to technological, training, and cultural barriers.^11^

To address these limitations, this study introduces a novel approach, demonstrating how online communities offer a complementary and valuable perspective to traditional health data, providing insights into the multimorbidity associated with diabetes, particularly in relation to mental health. We gathered data from Reddit (reddit.com), which is one of the most popular websites in the world,^12^ tapping into its diverse range of dedicated online communities, also known as subreddits. We manually confirmed that a wide range of physical and psychiatric conditions, including diabetes, have their own corresponding subreddits, each with a substantial number of active users. Using this online data, we mapped the multimorbidity of diabetes by linking subreddits with shared active users and constructed a multimorbidity network for both type 1 and type 2 diabetes. The study explores the following research questions:

1. What are the key multimorbidity patterns associated with diabetes, as reflected in the constructed multimorbidity network?
2. Can early signals of diabetes be detected by analyzing the temporal patterns of user participation across health-related online communities?

By addressing these research questions, this study contributes to the growing field of digital epidemiology and demonstrates the potential of online engagement data in understanding chronic disease progression. The findings provide valuable insights into the co-occurrence of diabetes with other health conditions, particularly mental health disorders, which are often underreported in clinical settings. Additionally, this study highlights the potential for online behavioral data to aid in the early detection of diabetes, which could inform public health strategies and personalized interventions.

## Methods

The Reddit data were collected from the Pushshift website^13^ for the top 40 000 subreddits in terms of user engagement (posts and replies) from 2005 to 2024. We focused on 3 primary diabetes-related subreddits, “diabetes,” “diabetes_t1,” and “diabetes_t2” and recorded 143 840 users in total (see Figure S1). Additionally, we manually identified subreddits associated with 88 other common diseases based on the items sourced from NHS Inform^14^ (see Table S1). A disease is considered correlated with diabetes if users of the 3 primary diabetes-related subreddits frequently engage in discussions within its corresponding subreddit.

It is important to point out that simply counting the number of users active in both diabetes-related and other disease-related subreddits may lead to misleading conclusions, as subreddit engagement does not necessarily indicate a personal connection to the disease. Users may participate for various reasons, such as caregiving responsibilities or general interest, rather than their own medical experiences. To address this, we apply statistical methods that account for chance co-occurrence and isolate only significant associations, ensuring that the identified relationships reflect meaningful patterns. In the following subsections, we describe our approach for testing the significance of subreddit links and detecting early signals of diabetes, respectively.

### Link significance

We created a detailed, weighted, and directed network of subreddits by tracing the engagement patterns of individual diabetic users across diverse health conditions for both type 1 and type 2 diabetes. Similar network analysis methods have been extensively utilized in other research fields, including studying disease correlations,^15^ tracking innovation substitutions,^16^ exploring scientific recognitions,^17^ and analyzing art institutes connections.^18^ By adopting this network-based approach, we mapped subreddit relationships to explore health-related correlations and identify potential early signals of diabetes. Specially, each subreddit was represented as a node with links formed based on shared user activity exceeding chance expectation. The network was built through a 3-step process:

1. *Calculating actual flow* **:** We quantified the actual flow between subreddits i and j ($W_{ij}$) by counting the total number of users engaged in both.
2. *Normalizing for chance* **:** To differentiate meaningful connections from those occurring randomly, we generated a user-subreddit bipartite network and shuffled links while preserving the engagement count per user and the user count per subreddit. This allowed us to compute the expected weight ${\tilde{W}}_{ij}$ for each subreddit pair.
3. *Establishing significant links* **:** We retained only subreddit pairs where the actual flow exceeds the expected value (${W_{ij}>\tilde{W}}_{ij}$), indicating a statistically significant connection.

Through this approach, we filtered out noise and uncovered meaningful patterns in user behavior across subreddits. Additionally, we performed community detection using the Louvain algorithm^19^ to map the positions and relationships of different subreddits within the network.

### Early signal detection

To systematically assess whether users engaged with other subreddits earlier than a given subreddit i, we introduce the *Temporal Probability Difference* (TPD) as a measure of early signal detection. The TPD quantified the deviation between the observed probability that subreddit j followed subreddit i (measured by the first instance of engagement in each subreddit) in a user’s engagement history ($P_{ij}$) and a corresponding baseline probability ($P_{ij}^{\prime }$). Formally, for each subreddit pair (i,j), we defined


$${\mathrm{TPD}}_{ij}=P_{ij}-P_{ij}^{'}.$$

The baseline probability $P_{ij}^{\prime }$ was derived through a bootstrapping process, where the engagement sequences of all users who co-engaged with i and j were randomly shuffled. Specifically, engagement instances were reassigned randomly across users while preserving the total number of instances for each subreddit. For each bootstrap iteration, we computed $P_{ij}^{\prime }$ and ${\mathrm{TPD}}_{ij}$, generating a distribution of TPD values for each subreddit pair. The mean of the distribution serves as the estimated TPD value, while the 95% confidence interval represented the uncertainty via error bars. A significant negative TPD value (below zero) indicates that the probability of subreddit j being engaged *after* subreddit i is significantly lower than expected. In other words, subreddit j systematically preceded subreddit i, suggesting that j may serve as an early signal for engagement with i.

## Results

### Mapping multimorbidity network

**Figure 1.**
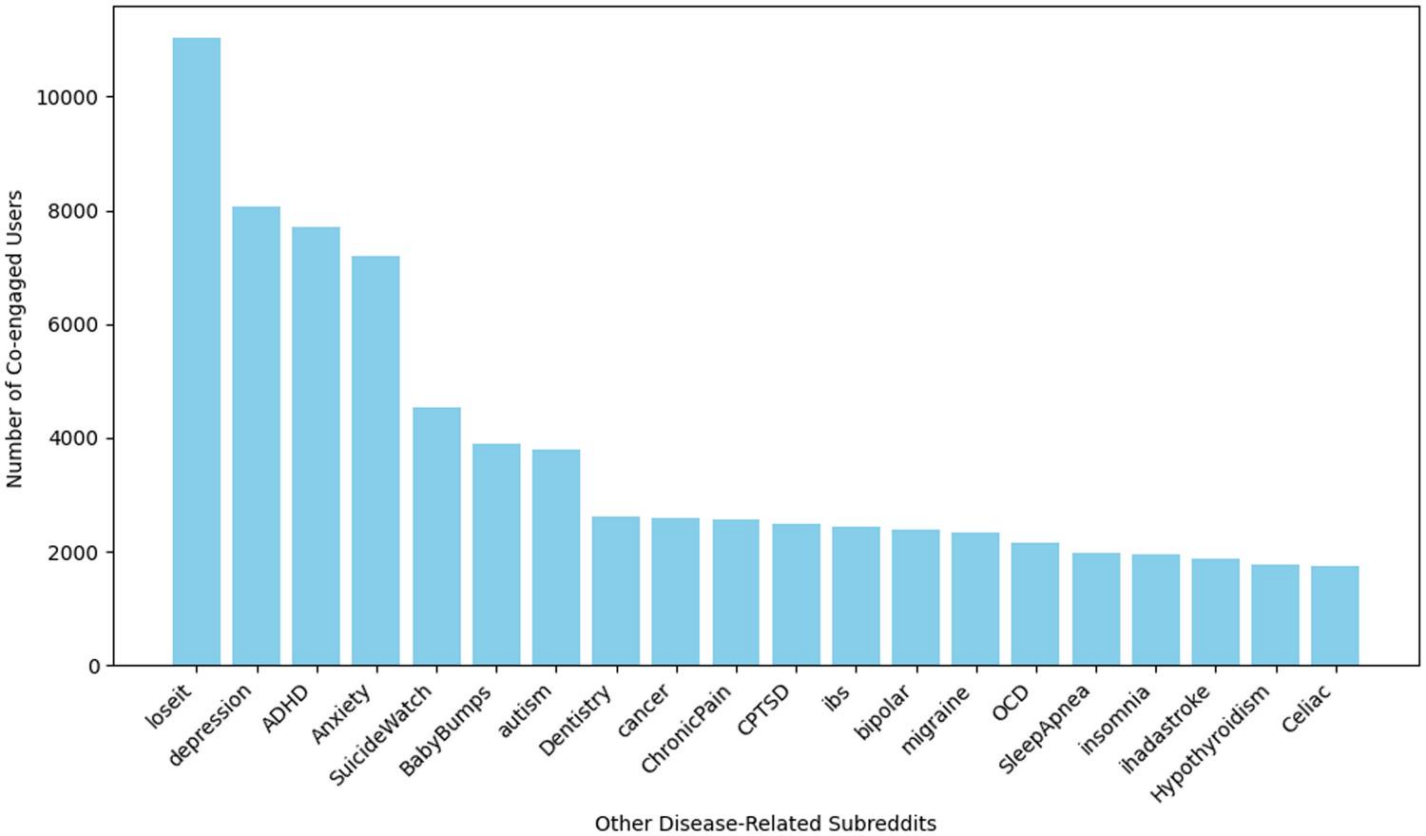
Top 20 subreddits most frequently co-engaged by users of diabetes-related subreddits, ordered by decreasing number of shared users. Prominent co-engagement with subreddits on weight loss (“loseit”) and mental health (“depression,” “ADHD,” “Anxiety,” “SuicideWatch”) highlights links between diabetes, obesity, and psychological burden. Other frequently co-engaged subreddits suggest broader multimorbidity concerns (eg, “BabyBumps,” “Dentistry,” “cancer,” “ChronicPain”).

To understand the multimorbidity of diabetes with the online data, we mapped the so-called multimorbidity network by tracing the engagement patterns of individual diabetic users across the other 88 subreddits. Based on a sample of 143 840 users with diabetes, Figure 1 illustrates how frequently users of diabetes-related subreddits also engage with other disease-related subreddits. It shows the top 20 correlated subreddits, ordered from left to right by decreasing number of co-engaged users.

In Figure 1, “loseit” appears as the most frequently co-engaged subreddit, suggesting that diabetes subreddit users are highly engaged with weight loss discussions, which aligns with the strong link between obesity and diabetes. Mental health-related subreddits such as “depression,” “ADHD,” “Anxiety,” and “SuicideWatch” also rank high, highlighting the significant psychological burden that may accompany diabetes. Other notable subreddits include “BabyBumps,” “Dentistry,” “cancer,” and “ChronicPain,” pointing to concerns about other potential multimorbidity health issues.

**Figure 2.**
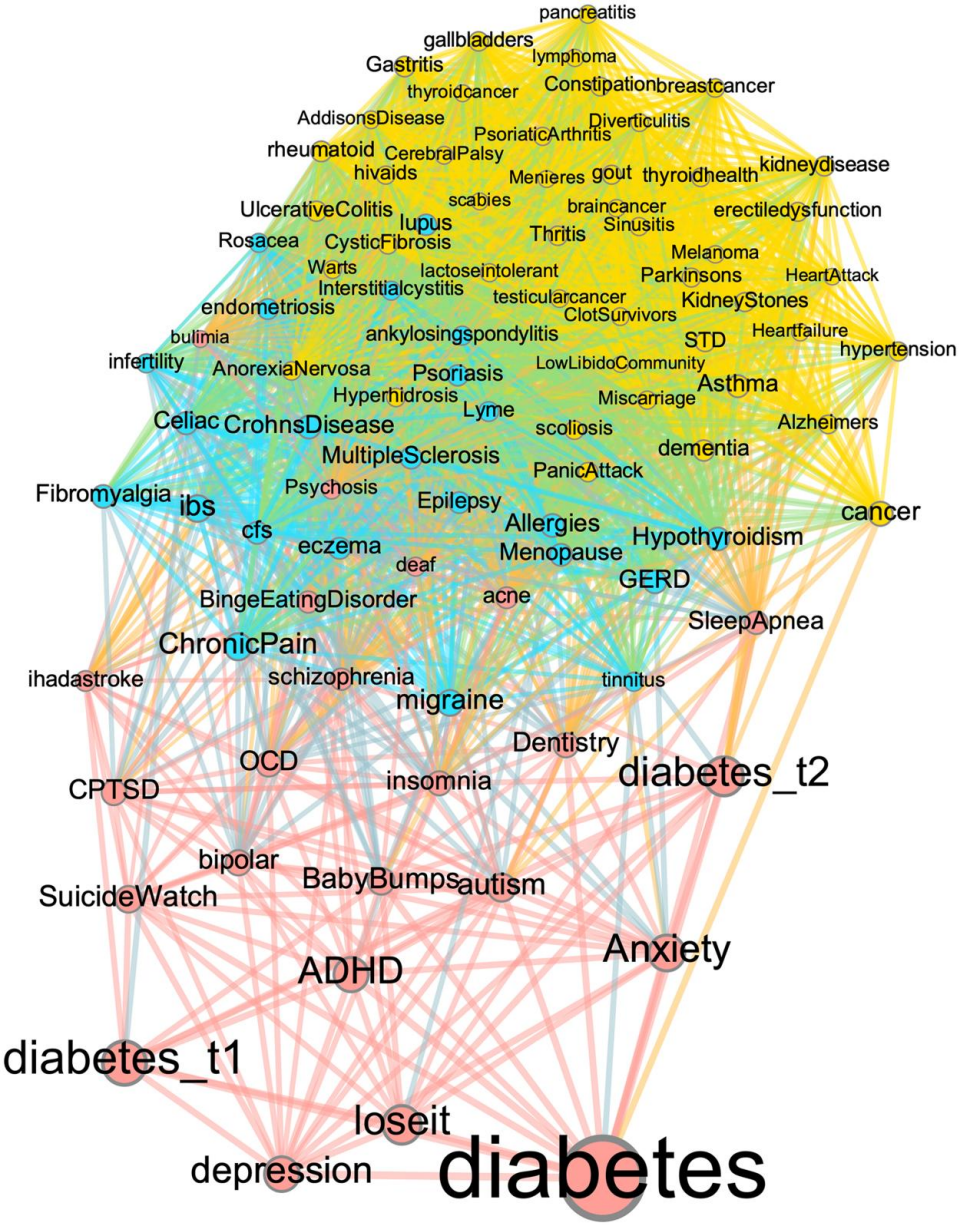
Multimorbidity network of diabetes constructed from subreddit co-engagement patterns. Nodes represent subreddits, sized by strength centrality, and colored by community detection. Edges indicate statistically significant user overlap beyond chance. Three main communities emerge: mental health (red), autoimmune and inflammatory conditions (blue), and chronic organ-specific conditions (yellow), illustrating the diverse multimorbidity landscape surrounding diabetes.

Following the methods outlined above, Figure 2 presents the multimorbidity network of diabetes, where nodes correspond to subreddits, and links are formed between 2 subreddits when the number of users co-engaging with them exceeds chance expectation. The size of each node (and its label) reflects its strength centrality while their color represents communities detected within the network (for details, see Methods).

As expected, since the analysis was based on users participating in the 3 primary diabetes-related subreddits, “diabetes” is the largest and most central node, with strong connections to its subcategories, “diabetes_t1” and “diabetes_t2.” Three main communities were detected in this network, represented by red, blue, and yellow respectively. The red community reveals a strong relationship between diabetes and mental health conditions. Subreddits such as “depression,” “Anxiety,” “ADHD,” “bipolar,” and “SuicideWatch” form a dense cluster around diabetes, suggesting that users who discuss diabetes also frequently engage in mental health discussions. This aligns with existing research showing that individuals with diabetes also experience mental health multimorbidity.^6–8^

The blue community consists of subreddits related to autoimmune and chronic conditions, including “ChronicPain,” “Fibromyalgia,” “MultipleSclerosis,” and “lupus.” These subreddits are tightly interconnected, indicating that users with one of these conditions are likely to participate in discussions about others. The blue community also includes gastrointestinal and allergic conditions such as “ibs,” “Celiac,” “eczema,” “GERD,” and “Hypothyroidism.” Together, these clustered subreddits suggest a shared user base among individuals managing autoimmune and inflammatory conditions alongside diabetes.^20^

Within the yellow community, subreddits center around organ-specific conditions such as “cancer,” “kidneydisease,” “pancreatitis,” and “Sinusitis,” reflecting long-term health concerns often discussed alongside diabetes. This cluster also highlights common multimorbidities including “hypertension” and various cardiovascular conditions such as “HeartAttack” and “Heartfailure,” which are well-documented comorbid risks for individuals with diabetes.^4^ In addition, the presence of subreddits like “gallbladders,” “KidneyStones,” and “thyroidhealth” points to frequent co-engagement related to urological and endocrine health.^5^

Overall, Figure 2 highlights the significant overlap in user participation across different health-related subreddits, emphasizing strong connections between diabetes and mental health, autoimmune diseases, gastrointestinal disorders, and reproductive health issues. These patterns suggest that online communities serve as valuable spaces for individuals managing multiple chronic conditions, reflecting both the psychological and physiological burdens associated with diabetes multimorbidity.

### Detecting early signals

**Figure 3.**
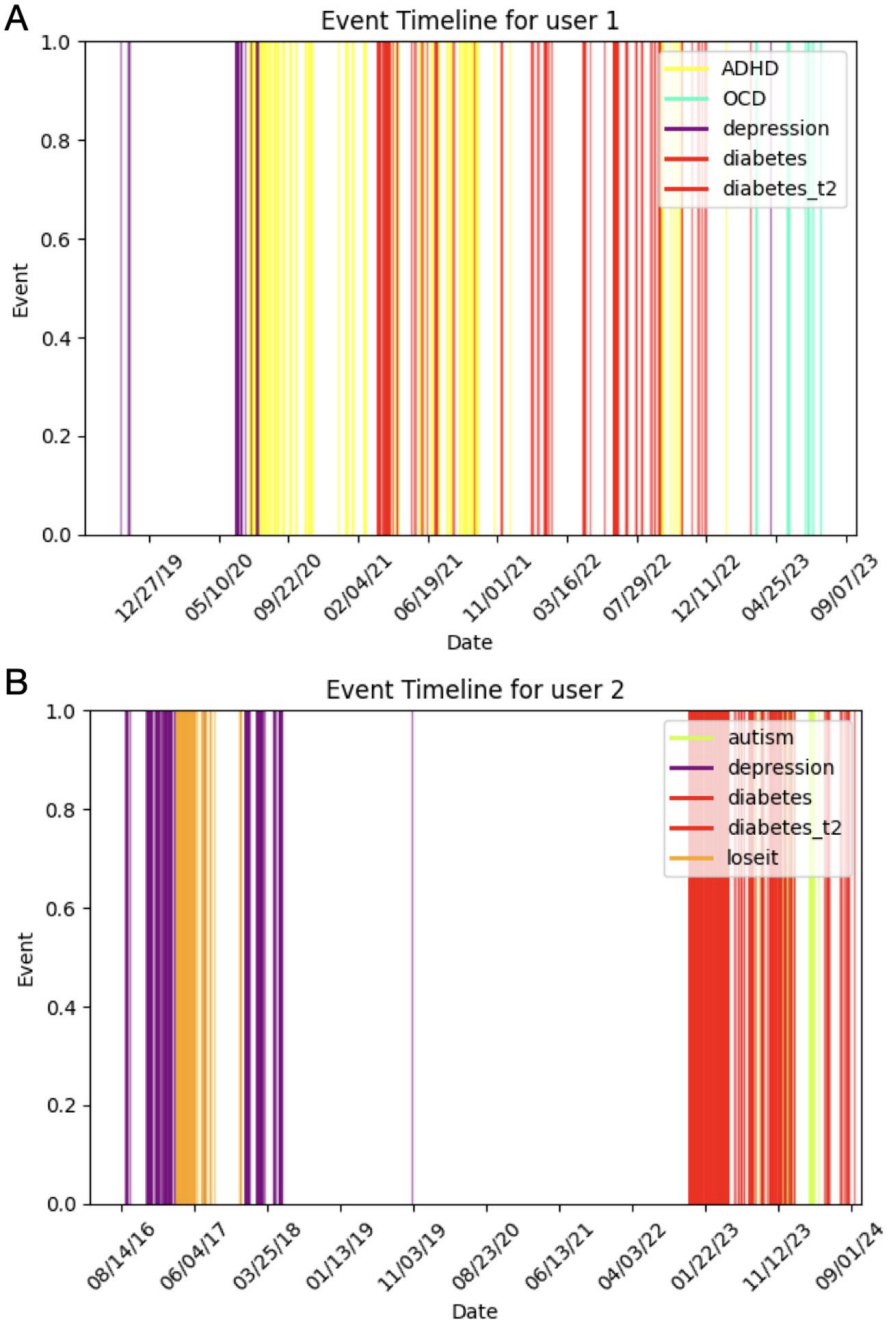
Two case studies illustrating potential early signals of diabetes through Reddit activity. (A) Shows a user engaging in mental health-related subreddits (“ADHD,” “depression”) prior to diabetes-related discussions. (B) Highlights a user active in both mental health (“depression”) and obesity-related (“loseit”) subreddits well before diabetes engagement. These patterns suggest that other conditions may precede and potentially signal the onset of diabetes.

Figure 3 presents 2 individual case studies illustrating the potential for identifying early signals of diabetes through Reddit activity. Figure 3A depicts a user whose participation in mental health-related subreddits (ie, “ADHD” and “depression”) predated engagement in diabetes-related discussions. The clustering of mental health-related activity prior to diabetes-related interactions suggests a possible temporal relationship, where individuals facing psychological challenges may later develop or receive a diagnosis for diabetes. This aligns with existing research indicating a bidirectional relationship between mental health disorders and metabolic conditions, where factors such as stress, medication side effects, and lifestyle choices influenced by mental health conditions could contribute to diabetes risk.

Figure 3B shows a different trajectory, where the user’s engagement with both a mental health-related subreddit (ie, “depression”) and an obesity-related subreddit (ie, “loseit”) appeared significantly before discussions in diabetes-related communities. This pattern suggests that weight management concerns might also serve as an early indicator of an eventual diabetes diagnosis. Given the well-documented link between obesity and type 2 diabetes, this finding reinforces the possibility that individuals attempting weight loss may already be on a path toward metabolic disorders. The gap between obesity-related discussions and diabetes-related engagement in this user’s timeline suggests that individuals may struggle with weight management long before being diagnosed or actively seeking support for diabetes. Together, these 2 case studies highlight the promise of using social media data to detect early behavioral markers of diabetes risk, potentially offering an avenue for health interventions.

**Figure 4.**
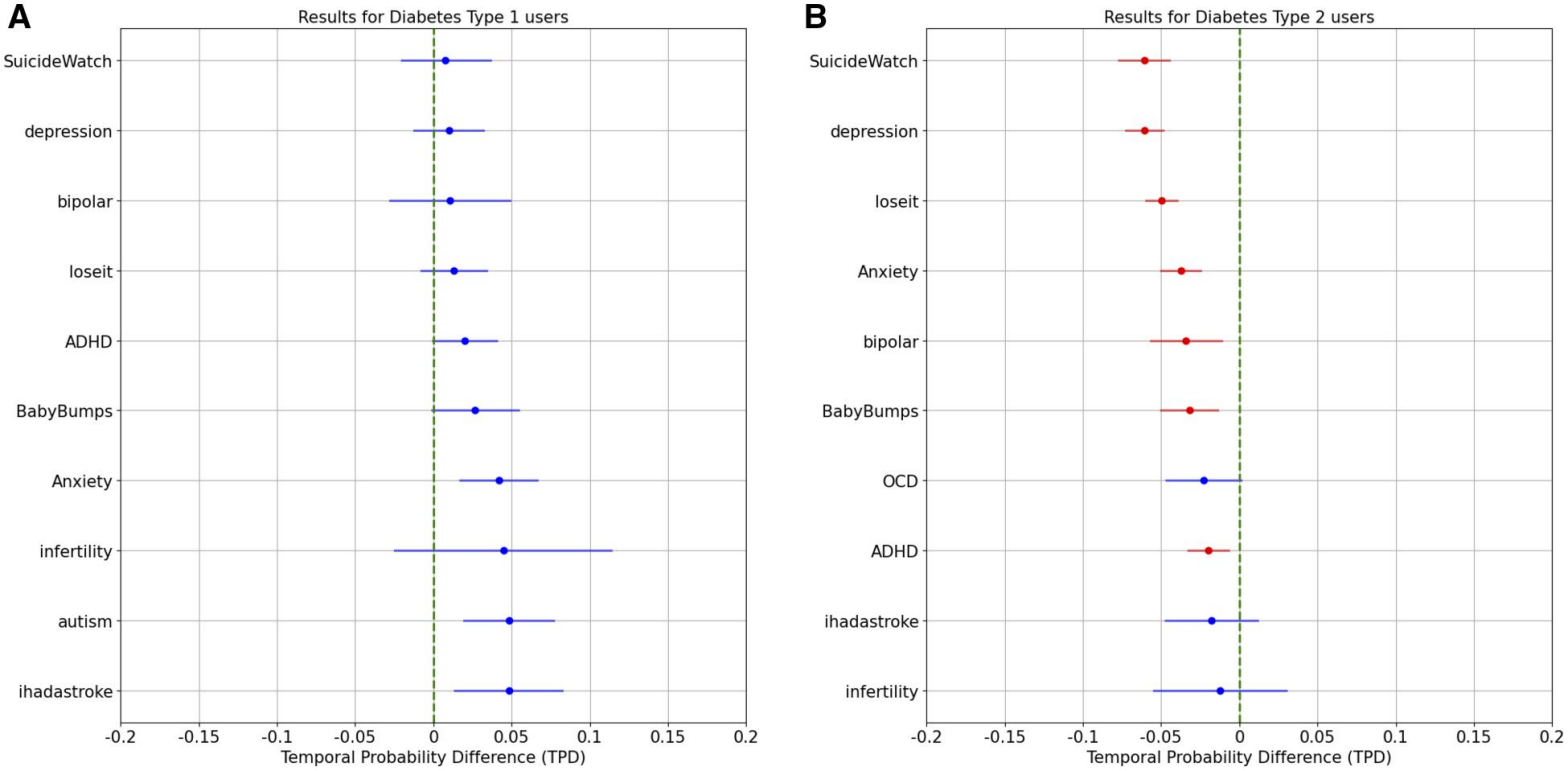
Statistical test results for early signals of diabetes based on TPD values. (A) Shows no significant early signals for type 1 diabetes. (B) Identifies significant early engagement in mental health, weight management, and pregnancy-related subreddits among type 2 diabetes users, suggesting these conditions may precede and signal risk of type 2 diabetes.

Based on separate samples of 46 585 users with type 1 diabetes and 99 287 users with type 2 diabetes, Figure 4 presents a formal statistical test for early signals, highlighting the 10 smallest TPD values for both types, respectively (see Figure S1 for definitions of the 2 user types, and Figures S2 and S3 for TPD values across all diseases). Figure 4A focuses on type 1 diabetes users and reveals no statistically significant early signals, as indicated by the confidence intervals overlapping with zero. This suggests that, within the dataset analyzed, there were no strong behavioral indicators on Reddit that reliably preceded a type 1 diabetes diagnosis. Given that type 1 diabetes is primarily an autoimmune condition often diagnosed in childhood or early adulthood,^21^ it is less likely to be preceded by observable behavioral patterns on Reddit.

Figure 4B, however, presents compelling evidence of early signals for type 2 diabetes. Several mental health-related subreddits (ie, “SuicideWatch,” “depression,” “Anxiety,” “bipolar,” and “ADHD”), weight management communities (ie, “loseit”), and pregnancy-related discussions (ie, “BabyBumps”) all show statistically significant temporal differences, indicating that engagement in these subreddits occurred before participation in diabetes-related discussions. Note that although “OCD,” “ihadastroke,” and “infertility” also have their TPD values below zero, these results are not statistically significant at the 95% level. This finding is particularly relevant given the established associations between mental health conditions, obesity, pregnancy, and type 2 diabetes. For example, depression and anxiety can influence eating behaviors and physical activity,^22^ while ADHD has been linked to impulsive eating and metabolic risks.^23^ Similarly, gestational diabetes has become a growing concern due to its rising prevalence and potential long-term health implications for both mothers and infants,^24^ making pregnancy another early signal of type 2 diabetes. These results reinforce the idea that digital behavioral data can be leveraged to identify individuals at higher risk of developing type 2 diabetes, potentially allowing for earlier interventions and support.

## Discussion

Diabetes is a chronic disease that affects millions of people worldwide and has increased its prevalence through lifestyle changes and globalization during the past decades.^25^ Patients with diabetes commonly experience multimorbidity, which can be defined as the co-occurrence of multiple chronic or acute diseases and medical conditions within one person.^1^^,^^2^ A better understanding of the nature, prevalence, and patterns of multimorbidity associated with diabetes can enhance patient management in primary care, enabling more patient-centered risk assessments and tailored treatments^26^ and lower economic costs.^27^ Previous studies have observed multimorbidity among both patients with type 1 diabetes^6^^,^^28^ as well as those with type 2 diabetes.^29^^,^^30^ However, existing analyses often rely on traditional health data such as electronic health records for specific cohorts.^31^^,^^32^ While these sources provide valuable insights, they lack comprehensive health information beyond healthcare facilities. This gap also means that opportunities for early warning or intervention may be compromised, as potential data only get recorded during patient interactions with healthcare providers.^9^

The recent availability of large-scale online datasets offers a unique opportunity to analyze individuals’ social footprints, providing unprecedented insights into their healthcare issues as well as interests and lifestyles.^33–35^ Consequently, there has been a surge in health research in recent years utilizing data sourced from popular online platforms such as Reddit.^36–41^ With the growing prevalence of these platforms, individuals living with diabetes increasingly turn to online communities for support, information, and connection.^42^^,^^43^ These platforms offer self-management education and peer support,^44^^,^^45^ as well as produce a wealth of user-generated data^46^ that can be used to trace health conditions related to diabetes, often extending beyond the reach of traditional health systems. Furthermore, this extensive online data present opportunities for detecting early signals of both mental and physical conditions that may precede the onset of diabetes.

Our contribution is at least threefold. First, we mapped the multimorbidity of diabetes using data from online communities, which was sourced from Reddit. It presents several advantages over traditional health data. On one hand, Reddit’s vast and diverse user base,^12^ drawn from across the globe, offers a broad representation of multimorbidity associated with diabetes on a large scale. On the other hand, the anonymity on this platform fosters a more open and candid discussion of health information and patient-centered experiences.^33^^,^^34^ Second, we constructed a detailed multimorbidity network for both type 1 and type 2 diabetes by tracing user activity across online communities focused on diabetes and related health conditions. The network science methodologies employed in this paper explicitly recognizes the interconnected nature of diabetes and its associated multimorbidity. Third, by analyzing temporal patterns in user activity, we identified obesity and mental health disorders as statistically significant early signals of type 2 diabetes, which holds promise for preventive health strategies targeting diabetes and its related conditions.

Despite these contributions, our study has limitations, particularly concerning the representativeness of Reddit users relative to the general population. While Reddit offers access to a vast repository of user-generated health discussions, its user base tends to skew toward younger, more technologically engaged individuals, potentially limiting the generalizability of our findings to broader, more demographically diverse populations. Moreover, the anonymity that fosters open and candid discourse also introduces challenges in verifying the authenticity and accuracy of self-reported health information. To address these limitations, future research could integrate online data with offline evidence, such as electronic health records or survey-based studies, to triangulate findings and enhance validity. Additionally, the use of advanced artificial intelligence techniques, particularly large language models (LLMs), holds promise for improving the quality of online health research. For instance, LLMs can be employed to automatically extract user attributes, assess the reliability of health claims, and detect inconsistencies or misinformation, ultimately increasing the robustness and interpretability of insights derived from online platforms.

## Conclusion

We analyzed multimorbidity patterns and early signals of diabetes in online communities using Reddit data. First, in the realm of healthcare, our findings provide valuable insights into the interconnected nature of diabetes and its associated multimorbidity, beyond what can be gleaned from traditional health data alone. Moreover, the application of network science methodologies to analyze data from online communities sets a precedent for future research. This innovative and interdisciplinary approach not only broadens the toolkit available to researchers but also highlights the potential of big data analytics in healthcare. As digital health data continue to grow, the study underlines the importance of leveraging these resources to extract meaningful knowledge that can drive advancements in medical science and clinical practice. Furthermore, given that Reddit encompasses information spanning both the health and social dimensions of individual users, the study holds promise for early detection and the development of interventions for diabetes and its related conditions.

## Supplementary Material

ooaf049_Supplementary_Data

## Data Availability

The code for link significance and early signal detection is publicly available in the GitHub repository https://github.com/chingjin/reddit_diabetes. The names and links of all disease-related subreddits are available in Table S1.
