# Supplementary material for: Multimorbidity patterns and early signals of diabetes in online communities
Source: JAMIA Open. 2025 May 30;8(3):ooaf049. doi: 10.1093/jamiaopen/ooaf049 (PMC12124401; doi:10.1093/jamiaopen/ooaf049)
Supplement: ooaf049_Supplementary_Data [file ooaf049_supplementary_data.docx]

Table A1. List of disease-related subreddits.

| **Disease Name** | **Subreddit Link** |
| --- | --- |
| Acne | <https://www.reddit.com/r/acne/> |
| Addison’s disease | <https://www.reddit.com/r/AddisonsDisease/> |
| Allergies | <https://www.reddit.com/r/Allergies/> |
| Alzheimer’s disease | <https://www.reddit.com/r/Alzheimers/> |
| Ankylosing spondylitis | <https://www.reddit.com/r/ankylosingspondylitis/> |
| Anorexia nervosa | <https://www.reddit.com/r/AnorexiaNervosa/> |
| Anxiety | <https://www.reddit.com/r/Anxiety/> |
| Asthma | <https://www.reddit.com/r/Asthma/> |
| Attention deficit hyperactivity disorder (ADHD) | <https://www.reddit.com/r/ADHD/> |
| Autistic spectrum disorder (ASD) | <https://www.reddit.com/r/autism/> |
| Binge eating | <https://www.reddit.com/r/BingeEatingDisorder/> |
| Bipolar disorder | <https://www.reddit.com/r/bipolar/> |
| Brain cancer | <https://www.reddit.com/r/braincancer/> |
| Breast cancer | <https://www.reddit.com/r/breastcancer/> |
| Bulimia | <https://www.reddit.com/r/bulimia/> |
| Cardiovascular disease | <https://www.reddit.com/r/Heartfailure/> |
| Cancer (general) | <https://www.reddit.com/r/cancer/> |
| Cerebral palsy | <https://www.reddit.com/r/CerebralPalsy/> |
| Chronic fatigue syndrome | <https://www.reddit.com/r/cfs/> |
| Chronic kidney disease | <https://www.reddit.com/r/kidneydisease/> |
| Chronic pain | <https://www.reddit.com/r/ChronicPain/> |
| Coeliac disease | <https://www.reddit.com/r/Celiac/> |
| Constipation | <https://www.reddit.com/r/Constipation/> |
| Coronary heart disease | <https://www.reddit.com/r/HeartAttack/> |
| Crohn’s disease | <https://www.reddit.com/r/CrohnsDisease/> |
| Cystic fibrosis | <https://www.reddit.com/r/CysticFibrosis/> |
| Cystitis | <https://www.reddit.com/r/Interstitialcystitis/> |
| Deep vein thrombosis | <https://www.reddit.com/r/ClotSurvivors/> |
| Dementia | <https://www.reddit.com/r/dementia/> |
| Depression | <https://www.reddit.com/r/depression/> |
| Diverticular disease and diverticulitis | <https://www.reddit.com/r/Diverticulitis/> |
| Eczema | <https://www.reddit.com/r/eczema/> |
| Endometriosis | <https://www.reddit.com/r/endometriosis/> |
| Epilepsy | <https://www.reddit.com/r/Epilepsy/> |
| Erectile dysfunction (impotence) | <https://www.reddit.com/r/erectiledysfunction/> |
| Fibromyalgia | <https://www.reddit.com/r/Fibromyalgia/> |
| Gallstones | <https://www.reddit.com/r/gallbladders/> |
| Gastro-oesophageal reflux disease (GORD) | <https://www.reddit.com/r/GERD/> |
| Gout | <https://www.reddit.com/r/gout/> |
| Hearing loss | <https://www.reddit.com/r/deaf/> |
| High blood pressure (hypertension) | <https://www.reddit.com/r/hypertension/> |
| HIV | <https://www.reddit.com/r/hivaids/> |
| Hyperhidrosis | <https://www.reddit.com/r/Hyperhidrosis/> |
| Insomnia | <https://www.reddit.com/r/insomnia/> |
| Irritable bowel syndrome (IBS) | <https://www.reddit.com/r/ibs/> |
| Kidney stones | <https://www.reddit.com/r/KidneyStones/> |
| Lactose intolerance | <https://www.reddit.com/r/lactoseintolerant/> |
| Loss of libido | <https://www.reddit.com/r/LowLibidoCommunity/> |
| Lupus | <https://www.reddit.com/r/lupus/> |
| Lyme disease | <https://www.reddit.com/r/Lyme/> |
| Lymphogranuloma venereum (LGV) | <https://www.reddit.com/r/STD/> |
| Lymphoma | <https://www.reddit.com/r/lymphoma/> |
| Meniere’s disease | <https://www.reddit.com/r/Menieres/> |
| Menopause | <https://www.reddit.com/r/Menopause/> |
| Migraine | <https://www.reddit.com/r/migraine/> |
| Miscarriage | <https://www.reddit.com/r/Miscarriage/> |
| Multiple sclerosis (MS) | <https://www.reddit.com/r/MultipleSclerosis/> |
| Obesity | <https://www.reddit.com/r/loseit/> |
| Obsessive compulsive disorder (OCD) | <https://www.reddit.com/r/OCD/> |
| Obstructive sleep apnoea | <https://www.reddit.com/r/SleepApnea/> |
| Oral diseases | <https://www.reddit.com/r/Dentistry/> |
| Overactive thyroid | <https://www.reddit.com/r/thyroidhealth/> |
| Pancreatitis | <https://www.reddit.com/r/pancreatitis/> |
| Panic disorder | <https://www.reddit.com/r/PanicAttack/> |
| Parkinson’s disease | <https://www.reddit.com/r/Parkinsons/> |
| Post-traumatic stress disorder (PTSD) | <https://www.reddit.com/r/CPTSD/> |
| Pregnancy and baby | <https://www.reddit.com/r/BabyBumps/> |
| Psoriasis | <https://www.reddit.com/r/Psoriasis/> |
| Psoriatic arthritis | <https://www.reddit.com/r/PsoriaticArthritis/> |
| Psychosis | <https://www.reddit.com/r/Psychosis/> |
| Reactive arthritis | <https://www.reddit.com/r/Thritis/> |
| Rheumatoid arthritis | <https://www.reddit.com/r/rheumatoid/> |
| Rosacea | <https://www.reddit.com/r/Rosacea/> |
| Scabies | <https://www.reddit.com/r/scabies/> |
| Schizophrenia | <https://www.reddit.com/r/schizophrenia/> |
| Scoliosis | <https://www.reddit.com/r/scoliosis/> |
| Sinusitis | <https://www.reddit.com/r/Sinusitis/> |
| Skin cancer (melanoma) | <https://www.reddit.com/r/Melanoma/> |
| Stillbirth | <https://www.reddit.com/r/infertility/> |
| Stomach ulcer | <https://www.reddit.com/r/Gastritis/> |
| Stroke | <https://www.reddit.com/r/ihadastroke/> |
| Suicide | <https://www.reddit.com/r/SuicideWatch/> |
| Testicular cancer | <https://www.reddit.com/r/testicularcancer/> |
| Thyroid cancer | <https://www.reddit.com/r/thyroidcancer/> |
| Tinnitus | <https://www.reddit.com/r/tinnitus/> |
| Ulcerative colitis | <https://www.reddit.com/r/UlcerativeColitis/> |
| Underactive thyroid | <https://www.reddit.com/r/Hypothyroidism/> |
| Warts and verrucas | <https://www.reddit.com/r/Warts/> |

Notes: The list of common illnesses and conditions is sourced from [NHS Inform](https://www.nhsinform.scot/illnesses-and-conditions/a-to-z/). We searched online and manually verified the subreddit associated with each common disease. In addition to the three primary diabetes-related subreddits, the table above includes 88 manually matched subreddits with a significant number of users (i.e., among the top 40,000 from 2005 to 2024).


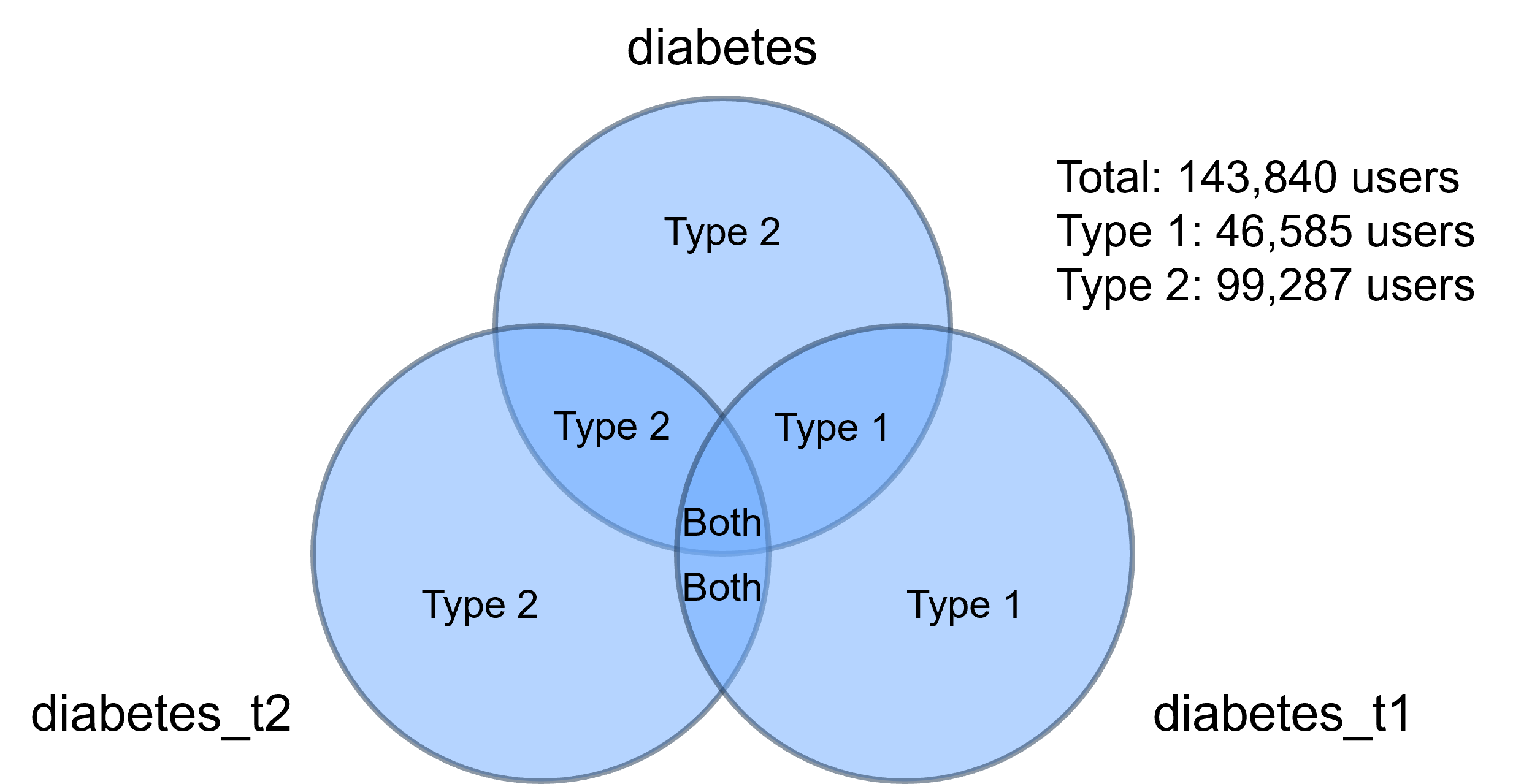


Figure A1. Definitions of Type 1 and Type 2 diabetes users from the three primary subreddits.


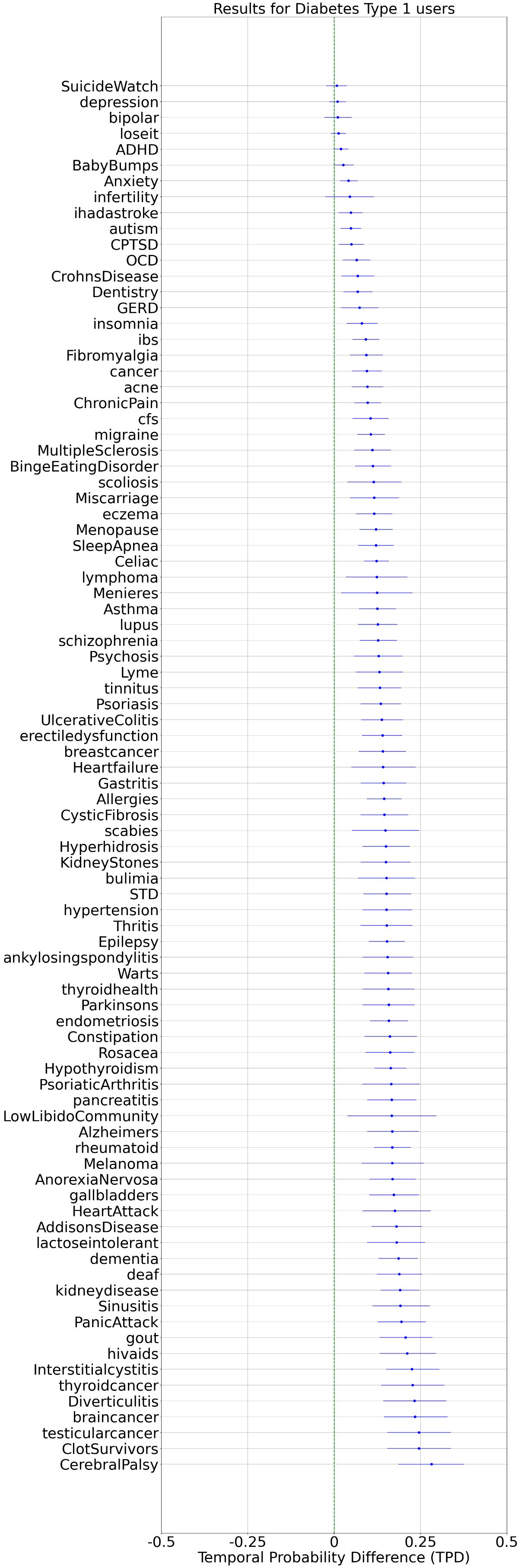


Figure A2. TPD between all diseases and Type 1 diabetes


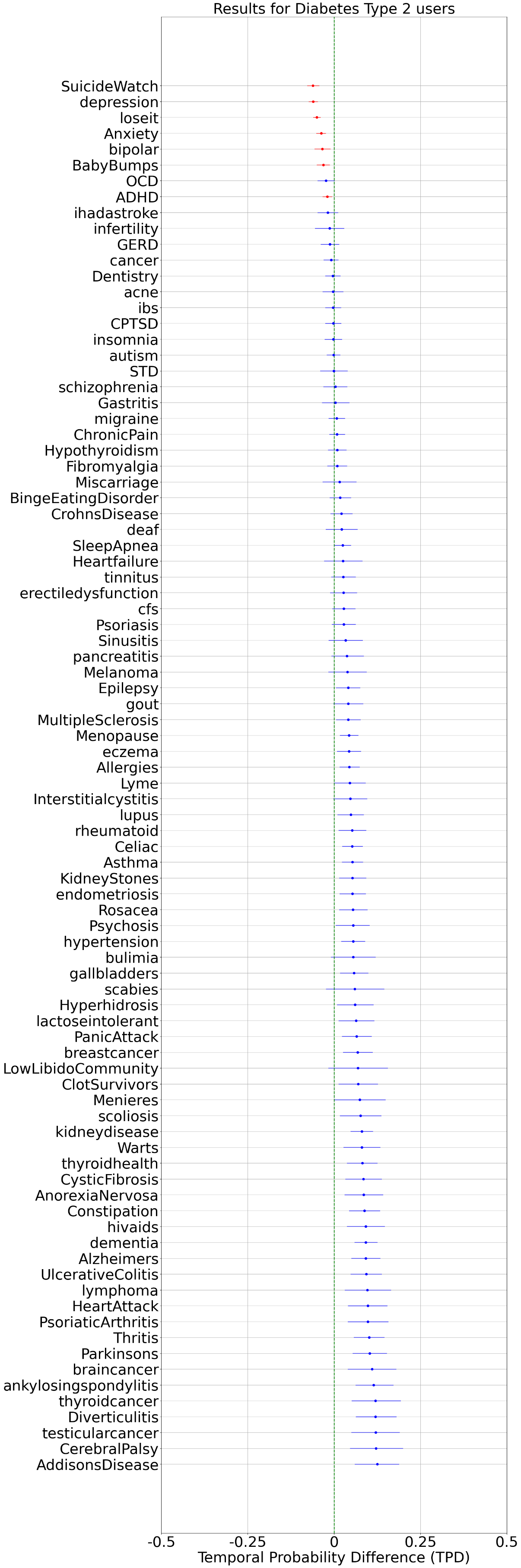


Figure A3. TPD between all diseases and Type 2 diabetes.
